# Integrating the Data From Microbiome and Metabolome Genome-Wide Association Studies to Uncover Gene-Microbe-Metabolite Interactions in Allergic Diseases

**DOI:** 10.7759/cureus.99600

**Published:** 2025-12-19

**Authors:** Yiwen Yuan, Yuwei Tang, Yu Sun

**Affiliations:** 1 College of Life Sciences, South China Agricultural University, Guangzhou, CHN

**Keywords:** allergic diseases, blood metabolomics, gene-microbiota-metabolite interactions, genome-wide association studies, gut microbiome

## Abstract

Background

Host genetics, gut microbiota, and metabolites have each been independently linked to allergic diseases such as asthma, allergic rhinitis, and eczema. However, the complex interactions between these three components remain poorly understood, largely due to a reliance on single-omics analyses. Integrating multi-omics data is essential for uncovering the underlying mechanisms of allergic disease pathogenesis.

Methodology

We performed a systematic, integrative analysis of large-scale public data from gut microbiome genome-wide association studies (GWAS) and blood metabolome-GWAS. We retrieved data from studies with cohorts of over 400 subjects. Overlapping genetic loci were identified by cross-referencing significant associations (p<1×10⁻⁶ for gene-microbe and p<1×10⁻⁵ for gene-metabolite) to define gene-microbe-metabolite trios. These trios were then cross-referenced with relevant databases (e.g., GWAS Catalog, gutMDisorder, and Human Metabolome Database (HMDB)) to establish their potential link to allergic diseases.

Results

Our integrative approach identified 12 distinct gene-gut microbiota-blood metabolite trios associated with allergic diseases. Established patterns were confirmed, including the ABO gene's influence on *Bifidobacterium bifidum*, which is known to impact immune regulation. Novel associations were also uncovered, including structural genes (e.g., LAMA2, PTPRT) potentially facilitating microbiota attachment and modulating metabolites such as octadecanedioate and genes involved in neurotransmitter signaling (e.g., SYN3, PDE1A), suggesting potential neuro-immune mechanisms.

Conclusions

By integrating microbiome-GWAS and metabolome-GWAS data, we have generated a valuable resource of candidate pathways underlying allergic disease pathogenesis. The identified trios provide specific, testable hypotheses for future validation studies and potential targets for biomarker discovery. This work underscores the power of multi-omics integration in allergy research and provides a clear roadmap for investigating complex gene-environment interactions.

## Introduction

Allergic diseases such as asthma, rhinitis, and eczema are complex conditions influenced by host genetics, environmental factors, and immune dysregulation [1-5]. Among environmental influences, the gut microbiome has emerged as an important modulator of immune development and allergic disease risk ​[6]. Alterations in the composition of the gut microbiota have been associated with asthma and other allergic conditions ​[6]. One mechanism by which the microbiome impacts the host immune system is through its metabolic products, such as short-chain fatty acids and other microbially derived metabolites, which can shape immune responses​ [7]. These observations suggest that host genes, commensal microbes, and metabolites may form interconnected networks influencing allergy outcomes.

Association studies have separately identified host genetic variants, gut microbiota, and blood metabolites that affect the occurrence of allergic diseases [2,8,9]. However, most existing research has focused on pairwise associations (e.g., gene-disease or microbe-disease), and a direct integration of these datasets to elucidate trio-level gene-microbe-metabolite interactions remains lacking. This gap is largely due to the predominance of single-omics studies and limited sample sizes (often 100-300 subjects) in multi-omics analyses, constraining statistical power to detect meaningful interactions [10]. Recent large-scale microbiome genome-wide association studies (GWAS) and metabolome-GWAS, with cohorts exceeding thousands of subjects, now enable such integrative investigations [11,12]. In this study, we applied a top-down approach by screening connected gene-microbe-metabolite trios from these studies to identify potential pairs that may contribute to allergic diseases. The primary objective of this study was to systematically identify and prioritize gene-microbe-metabolite trios with plausible mechanistic links to allergic diseases. By leveraging large-scale GWAS resources, we demonstrate that understanding gene-microbe-metabolite interactions provides a more comprehensive view of allergic disease pathogenesis and helps identify potential biomarkers for diagnosis or therapeutic targets.

## Materials and methods

**Figure 1:**
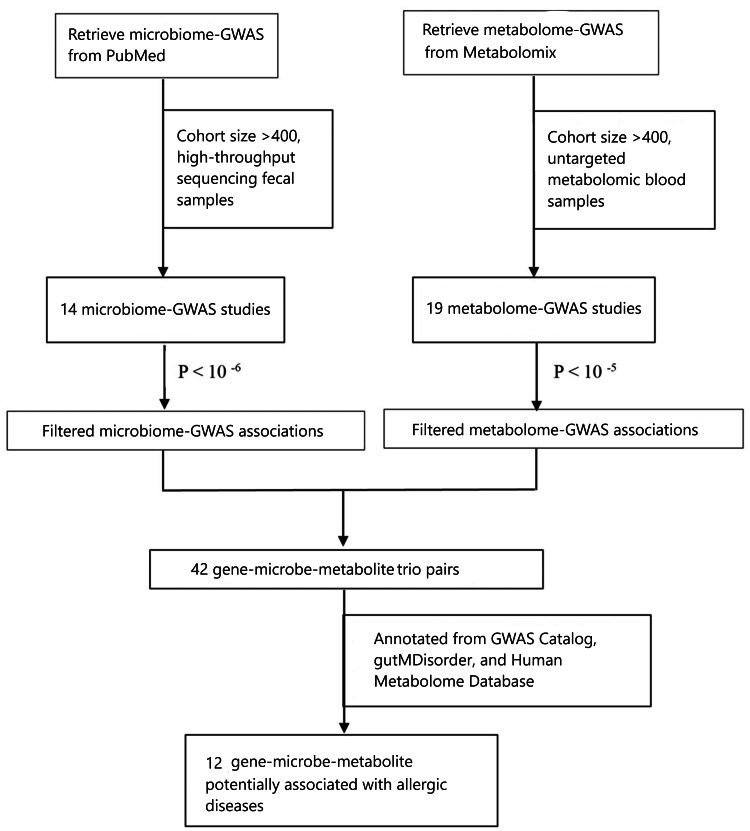
Workflow for the integrative analysis of GWAS summary statistics The schematic illustrates the multi-step process used to identify gene-microbe-metabolite trios associated with allergic diseases. The workflow began with a systematic search and selection of relevant microbiome-GWAS and metabolome-GWAS studies, followed by data extraction and identification of overlapping genetic loci. The final step involved biological annotation and literature review to prioritize trios with plausible links to allergy pathogenesis. GWAS: genome-wide association studies

Our study employed a systematic, multi-step approach to integrate publicly available GWAS summary statistics and identify gene-microbe-metabolite trios relevant to allergic diseases. The workflow is summarized in Figure 1.

Systematic literature search and study selection

To identify relevant datasets, we conducted a systematic literature search in PubMed and the Metabolomix database (http://www.metabolomix.com/) for studies published between January 2012 and December 2024. The PubMed search utilized terms including ("GWAS" OR "genome-wide association study") AND ("microbiome" OR "microbiota" OR "gut"). The Metabolomix database, a curated repository of metabolome-GWAS studies, was searched for all available blood metabolome studies. Studies were included if they met the following criteria: (1) performed a GWAS on either the gut microbiome or blood metabolome, (2) involved human subjects, (3) had a cohort size exceeding 400 participants to ensure adequate statistical power, and (4) utilized high-throughput methods for omics profiling (e.g., 16S rRNA or shotgun metagenomic sequencing for microbiome; mass spectrometry-based platforms for metabolomics). Two authors (Y.Y. and Y.T.) independently screened titles and abstracts, with any discrepancies resolved by consensus. This process yielded 14 microbiome-GWAS [11,13-25] and 18 metabolome-GWAS studies [26-43] for inclusion in our analysis (Table 1). The combined total sample size across all included studies exceeded 130,000 participant observations.

**Table 1:** Summary of gut microbiome-GWAS and blood metabolome-GWAS studies utilized for data analysis The table lists the 14 microbiome and 18 metabolome cohorts included in the integrative analysis. References for each study are provided in the Authors column. GWAS: genome-wide association studies; MS: mass spectrometry

No.	Publication	Authors	Year of publication	Sequencing method	Cohort size	Population
Gut microbiome-GWAS						
1	The interplay between host genetics and the gut microbiome reveals common and distinct microbiome features for complex human diseases	Xu et al. [13]	2020	16S	1475	Chinese
2	Mendelian randomization analyses support causal relationships between blood metabolites and the gut microbiome	Liu et al. [14]	2022	Shotgun metagenomics	3432	Chinese
3	A genome-wide association study for gut metagenome in Chinese adults illuminates complex diseases	Liu et al. [15]	2021	Shotgun metagenomics	1295	Chinese
4	Environment dominates over host genetics in shaping human gut microbiota	Rothschild et al. [16]	2018	16S	1046	Israeli
5	The effect of host genetics on the gut microbiome	Bonder et al. [17]	2016	Shotgun metagenomics	1514	Dutch
6	Effect of host genetics on the gut microbiome in 7,738 participants of the Dutch Microbiome Project	Lopera-Maya et al. [18]	2022	Shotgun metagenomics	7738	Dutch
7	Genetic determinants of the gut microbiome in UK twins	Goodrich et al. [19]	2016	16S	1126	British
8	A comprehensive assessment of demographic, environmental, and host genetic associations with gut microbiome diversity in healthy individuals	Scepanovic et al. [20]	2019	16S	858	European
9	Combined effects of host genetics and diet on human gut microbiota and incident disease in a single population cohort	Qin et al. [21]	2022	Shotgun metagenomics	5959	Finnish
10	Large-scale association analyses identify host factors influencing human gut microbiome composition	Kurilshikov et al. [11]	2021	16S	18340	Multi-ethnic
11	Genome-wide association study in 8,956 German individuals identifies influence of ABO histo-blood groups on gut microbiome	Rühlemann et al. [22]	2021	16S	8956	German
12	Genome-wide association analysis identifies variation in vitamin D receptor and other host factors influencing the gut microbiota	Wang et al. [23]	2016	16S	1812	German
13	Genome-wide associations of human gut microbiome variation and implications for causal inference analyses	Hughes et al. [24]	2020	16S	3890	Belgian, German
14	Association of host genome with intestinal microbial composition in a large healthy cohort	Turpin et al. [25]	2016	16S	1561	Canadian
Blood metabolome-GWAS						
1	Whole genome association study of the plasma metabolome identifies metabolites linked to cardiometabolic disease in black individuals	Tahir et al. [26]	2022	Non-targeted MS	2466	African American
2	Genomic atlas of the plasma metabolome prioritizes metabolites implicated in human diseases	Chen et al. [27]	2023	Non-targeted MS	8299	Canadian
3	Rare and common genetic determinants of metabolic individuality and their effects on human health	Surendran et al. [28]	2022	Non-targeted MS	14296	European
4	Ratios of acetaminophen metabolites identify new loci of pharmacogenetic relevance in a genome-wide association study	Thareja et al. [29]	2022	Non-targeted MS	520	Qatari
5	Whole-exome sequencing identifies rare genetic variants associated with human plasma metabolites	Bomba et al. [30]	2022	Non-targeted MS	3924	British
6	Genome-wide association studies of metabolites in Finnish men identify disease-relevant loci	Yin et al. [31]	2022	Non-targeted MS	6136	Finnish
7	Metabolome genome-wide association study identifies 74 novel genomic regions influencing plasma metabolites levels	Hysi et al. [32]	2022	Non-targeted MS	8809	European
8	Genome-wide association study of serum metabolites in the African American Study of Kidney Disease and Hypertension	Luo et al. [33]	2021	Non-targeted MS	619	African American
9	Unique genetic architecture of CSF and brain metabolites pinpoints the novel targets for the traits of human wellness	Wang et al. [34]	2023	Non-targeted MS	1028	Chinese
10	Genome-wide association and Mendelian randomization analysis prioritizes bioactive metabolites with putative causal effects on common diseases	Qin et al. [35]	2020	Non-targeted MS	7013	Finnish
11	A genome-wide association study discovers 46 loci of the human metabolome in the Hispanic Community Health Study/Study of Latinos	Feofanova et al. [36]	2020	Non-targeted MS	3926	Hispanic
12	Metabolic GWAS of elite athletes reveals novel genetically-influenced metabolites associated with athletic performance	Al-Khelaifi et al. [37]	2019	Non-targeted MS	490	International elite athletes
13	Whole-exome sequencing identifies common and rare variant metabolic QTLs in a Middle Eastern population	Yousri et al. [38]	2018	Non-targeted MS	614	Qatari
14	Mining the unknown: a systems approach to metabolite identification combining genetic and metabolic information	Krumsiek et al. [39]	2012	Non-targeted MS	1768	German
15	An atlas of genetic influences on human blood metabolites	Shin et al. [40]	2014	Non-targeted MS	7824	European
16	Genetic influences on metabolite levels: a comparison across metabolomic platforms	Yet et al. [41]	2016	Non-targeted MS	1001	British
17	Whole-genome sequencing identifies common-to-rare variants associated with human blood metabolites	Long et al. [42]	2017	Non-targeted MS	1960	British
18	Genetic studies of urinary metabolites illuminate mechanisms of detoxification and excretion in humans	Schlosser et al. [43]	2020	Non-targeted MS	5023	German

Data extraction and filtering

For each included study, we obtained the publicly available summary-level association statistics. We extracted the following information for all reported significant associations: SNP identifier (rsID), chromosome, genomic position (human genome build GRCh37/hg19), effect allele, p-value, and the associated microbial taxon or metabolite. To balance discovery with a high-confidence signal, we applied suggestive significance thresholds commonly used in quantitative trait loci (QTL) studies. We identified significant associations using a threshold of p<1×10⁻⁶ for gene-microbe associations and p<1×10⁻⁵ for gene-metabolite associations. These thresholds are more lenient than the strict genome-wide significance level (p<5×10⁻⁸) to allow for the identification of pleiotropic loci that might not reach that stringent level for both traits simultaneously in this exploratory analysis.

Identification of overlapping genetic loci

The core of our method was to identify exact variant-level overlaps or high linkage disequilibrium (LD) proxies associated with both a microbial taxon and a blood metabolite. Overlapping loci were defined by the presence of either the same lead SNP in both a microbiome-GWAS and a metabolome-GWAS or two different SNPs in high LD. To assess LD, we used the 1000 Genomes Project European (EUR) population panel as a reference. For each significant SNP identified in one GWAS type, we searched for significant SNPs in the other GWAS type that were located within a 250 kb window and exhibited an LD of r²>0.8. For each identified overlapping locus, we recorded the implicated microbial taxon and metabolite. Candidate genes within these loci were annotated using the Ensembl genome browser, prioritizing the nearest protein-coding gene or a gene previously linked to the trait in the literature.

Biological annotation and prioritization

Finally, each identified gene-microbe-metabolite trio was evaluated for its potential relevance to allergic diseases. We conducted a literature review and searched established databases, including the GWAS Catalog (https://www.ebi.ac.uk/gwas/), gutMDisorder (http://bio-computing.hrbmu.edu.cn/gutMDisorder/), and Human Metabolome Database (https://hmdb.ca/). Trios were prioritized for inclusion in our final results if the gene, microbe, or metabolite had previously been implicated in immune function, inflammation, or allergic conditions such as asthma, rhinitis, or eczema.

## Results

Our systematic cross-comparison of 14 microbiome-GWAS and 19 metabolome-GWAS datasets revealed 12 unique genetic loci with pleiotropic effects on both gut microbiota and metabolites that are plausibly linked to allergic diseases. The complete list of these gene-microbe-metabolite trios, including associated statistics and annotations, is presented in Table 2.

**Table 2:** Gene-microbe-metabolite pairs potentially associated with allergic diseases Genes, microbes, and metabolites that have been previously reported in association with allergic diseases are highlighted in bold and followed by an asterisk (*). SNPs and P-values for microbiome-GWAS and metabolome-GWAS associations are presented. GWAS: genome-wide association studies

Traits	Genes	Gene function	Microbiome GWAS			Metabolome GWAS		
			SNPs (location)	Gut microbes	P-value	SNPs (location)	Blood metabolites	P-value
Allergic diseases (asthma, rhinitis, and eczema)	ABO*	Glycosphingolipid biosynthesis	rs687289 (intron)	Bifidobacterium bifidum	8.95E-14	rs532436 (intron)	Campesterol	7.69E-13
	SYN3	Neurotransmitter release and synaptic function	rs763101897 (intron)	Ruminococcus gnavus*	5.33E-07	rs2008206 (intron)	Succinylcarnitine	5.03E-06
Asthma	CAMK4*	Calcium-dependent signaling	rs367882553 (intron)	Dorea longicatena	5.94E-07	rs6875225 (intron)	4-Cholesten-3-one	9.08E-06
	LAMA2*	Tissue structure scaffolding	rs139280910 (intron)	Bifidobacterium angulatum	7.2E-09	rs265369 (intron)	Octadecanedioate	1.24E-07
	PTPRT*	Regulates cellular processes including growth and differentiation	rs6072799 (intron)	Bifidobacterium angulatum	5.52E-07	rs6030381 (intron)	Octanoylcarnitine	1.97E-07
	SLC9A9*	A sodium/hydrogen exchanger/transporter	rs11371772 (intron)	Neisseria gonorrhoeae	3.26E-07	rs12488603 (intron)	N-oleoyltaurine	9.86E-06
	TENM3*	Neuronal development, immune regulation, and inflammation	rs552913575 (intron)	Granulicatella adiacens	7.73E-07	rs7694663 (intron)	Isoeugenol sulfate	8.91E-06
Eczema	AGAP1	Intracellular trafficking, endocytosis	rs146139464 (intron)	Eggerthella lenta*	7.45E-07	rs2675124 (intron)	3-Phenylpropionate	4.75E-06
	LUZP2	Cytoskeleton and cellular shape	rs61885278 (intergenic)	Prevotella buccae*	3.5E-08	rs2716449 (intron)	Choline phosphate	1.06E-06
	PDE1A	Cellular signaling, regulating smooth muscle contraction	rs1467463821 (intron)	Megasphaera micronuciformis*	3.60E-07	rs1432523 (intron)	Lactosyl-N-behenoyl-sphingosine	2.61E-06
	ROBO2	Axon guidance	rs4683988 (intron)	Bacteroides clarus*	8.79E-07	rs2593865 (intron)	Glycosyl ceramide	2.32E-08
	SPOCK3	Regulating extracellular matrix and cell-matrix interactions	rs575866680 (intron)	Megasphaera micronuciformis*	5.18E-08	rs56258049 (intron)	N-acetyl-aspartyl-glutamate	5.81E-06

The identified trios can be broadly categorized based on the function of the host gene, revealing several distinct biological themes. One prominent category involves genes influencing cellular structure and host-microbe interaction, exemplified by the link between the ABO gene and *Bifidobacterium bifidum *and the LAMA2 gene and *Bifidobacterium angulatum*. A second key theme is neuro-immune signaling, highlighted by associations involving SYN3 with *Ruminococcus gnavus *and PDE1A with *Megasphaera micronuciformis*, pointing to a potential gut-brain axis link in allergic inflammation. A third category involves genes regulating cellular processes and immune function, including PTPRT and TENM3, which were associated with *Bifidobacterium angulatum *and *Granulicatella adiacens*, respectively. These thematic groupings provide a framework for understanding the diverse biological pathways through which host genetics may interact with the microbiome and metabolome to influence allergy risk.

## Discussion

The identification of the established ABO*-Bifidobacterium bifidum* interaction serves as a crucial positive control, supporting the validity of our integrative approach [44,45]. The ABO gene influences gut glycosylation, producing specific glycan structures that serve as binding sites or nutrients for bacteria like *B. bifidum* [46]. By modulating the abundance of beneficial bacteria, ABO may influence the risk of allergic diseases. This finding illustrates how the gut microbiota can act as a biologically plausible mediator between host genetic factors and disease outcomes.

Beyond established associations, we uncovered novel patterns involving genes not previously linked to allergic diseases through this multi-omics lens. A prominent theme involved genes related to cellular structure and the extracellular matrix, including LAMA2, PTPRT, and SPOCK3. These genes may facilitate gut microbiota attachment to host tissues. For instance, the trio linking the LAMA2 gene, *Bifidobacterium angulatum*, and the metabolite octadecanedioate suggests a mechanism where host genetics influences epithelial integrity, shaping colonization by beneficial microbes that in turn produce anti-inflammatory metabolites. This hypothesis regarding the importance of the cellular matrix is supported by a recent radiomultiomic study in severe asthma patients [47].

Additionally, genes involved in neurotransmitter signaling, such as SYN3 and PDE1A, indicate a potential neuro-immune connection in asthma and eczema. Specifically, the interaction between SYN3, the pro-inflammatory gut microbe *Ruminococcus gnavus*, and succinylcarnitine points to a functional gut-brain axis link in modulating allergic inflammation. SYN3 may influence gut function via neural signaling, affecting colonization by *R. gnavus*, which in turn alters carnitine metabolism, a pathway previously implicated in the pro-inflammatory landscape of inflammatory bowel disease [48].

Strengths and limitations

A key strength of this study is its integrative, top-down approach that leverages the statistical power of multiple large-scale public datasets. While individual multi-omics cohort studies are invaluable, they are often limited by sample size, which constrains the power to detect significant interactions. By starting with robustly significant GWAS associations from cohorts totaling tens of thousands of individuals, our method effectively filters for high-confidence genetic loci with pleiotropic effects. This approach provides a computationally efficient and powerful framework for generating a prioritized list of biologically plausible and testable hypotheses. Furthermore, the identification of the well-established ABO*-Bifidobacterium* interaction serves as a positive control, validating the soundness of our integrative methodology and lending confidence to the novel associations we uncovered.

We acknowledge several limitations of this study. First, our analysis relied on summary statistics from different GWAS, which inherently limits the resolution of the findings and precludes individual-level data checks, necessitating the assumption of comparable population structures. Additionally, the cross-sectional nature of standard GWAS designs restricts the ability to infer temporal causality. We could only consider taxa and metabolites that were analyzed in those GWAS and reached significance thresholds. Consequently, important gene-microbe or gene-metabolite relationships might have been missed if they did not surpass genome-wide significance individually, yet they could still be biologically meaningful. Second, the taxonomic resolution in the microbiome-GWAS was at the genus level for many associations; finer strain-level or functional insights into the microbiota were not available, which means the actual microbial effector (e.g., a specific species or gene function) remains uncertain. Third, while we identified statistical overlaps, we cannot confirm causality or directionality of the interactions with this approach. It is unclear whether the host genetic variant affects the metabolite via the microbiota or independently influences both traits in parallel. Mendelian randomization or colocalization analyses could be applied in future studies to help distinguish mediation from pleiotropy. Finally, our focus was on allergic disease implications, but the data itself were not stratified by case-control status for any allergy. Therefore, we infer relevance to allergy indirectly based on known literature links; dedicated studies incorporating allergy phenotypes (e.g., GWAS of allergy risk combined with these data layers) would be needed to directly establish each interaction's contribution to disease.

Future directions and clinical implications 

The findings presented here provide a clear roadmap for subsequent research. The most critical next step is the functional validation of the novel trios. For instance, using in vitro models such as gut epithelial co-cultures, researchers could investigate whether genetic variants in LAMA2 alter the adhesion of *Bifidobacterium angulatum* and subsequently modulate metabolite production. Furthermore, advanced statistical methods like two-step Mendelian randomization could be applied to dissect the causal relationships within these trios, for example, to test whether the effect of a host genetic variant on a metabolite is mediated through its effect on a specific microbe.

From a clinical perspective, these trios hold significant potential. If validated, they could serve as multi-omics biomarkers for identifying individuals at high risk of developing allergic diseases. A composite panel measuring a patient's genetic variant, the abundance of a specific microbe, and the level of a key metabolite could offer far greater predictive power than any single marker. Ultimately, this work could pave the way for personalized interventions. For example, an individual with a risk variant in a gene affecting gut glycosylation might be advised to take specific prebiotics or probiotics to promote the growth of beneficial bacteria, thereby mitigating their genetic predisposition to allergy.

## Conclusions

We demonstrate that integrating microbiome-GWAS and metabolome-GWAS data is a promising strategy to uncover gene-microbe-metabolite interactions that may underlie complex diseases such as allergies. This short report provides proof of concept that leveraging existing large datasets can generate new hypotheses about biological pathways connecting host genetics, commensal microbes, and metabolic immune modulation. The identified gene-microbe-metabolite pairs point to specific mechanisms that could be driving allergic inflammation, offering potential biomarkers and intervention points. Future research should validate these findings in clinical cohorts and experimental models, which will be crucial for translating these insights into therapeutic interventions. Our approach highlights the power of multi-omics integration in illuminating the intricate network of factors at play in allergic disease and sets the stage for more comprehensive analyses as additional datasets become available.
